# Supplementary material for: Conformations and sequence determinants in the lipid binding of an adhesive peptide derived from Vibrio cholerae biofilms
Source: PLoS Pathog. 2026 Feb 19;22(2):e1013990. doi: 10.1371/journal.ppat.1013990 (PMC12965690; doi:10.1371/journal.ppat.1013990)
Supplement: S1 Table — (DOCX) [file ppat.1013990.s010.docx]

| **Strain Name in Manuscript** | **Genotype and Antibiotic Resistance** | **Description** | **Strain#**  **& Reference** |
| --- | --- | --- | --- |
| Rg background | *vpvC*^W240R^*,* Sm^R^ | Missense mutation in the *Vibrio cholerae* O1 El tor strain that elevates the level of cyclic-di-GMP. Rugose phenotype. Serves as the parental strain for most of the mutants in this manuscript | JY028 [4] |
| Δ*rbmC* | *vpvC*^W240R^*,* Δ*rbmC,* ∆*VC1807*::*P_tac_-mNeonGreen*, Spec^R^ | Clean deletion of *rbmC* by cotransformation with ∆*VC1807*::*P_tac_-mNeonGreen,* Spec^R^ | ZJ033 [5] |
| Δ*rbmC* Δ*bap1* | *vpvC*^W240R^*,* Δ*bap1,* Δ*rbmC,* ∆*VC1807*::*P_tac_-mNeonGreen*, Spec^R^ | Clean deletion of *rbmC* by cotransformation with ∆*VC1807*::*P_tac_-mNeonGreen,* Spec^R^ into a rugose strain lacking *bap1* (JY074) | STN0009 [6] |
| Δ*rbmC bap1*_Δ_*_57aa_* | *vpvC*^W240R^*,* Δ*rbmC, bap1*_Δ_*_57aa_,* ∆*VC1807*::*P_tac_-mNeonGreen,* Spec^R^ | Replacement of *bap1^WT^* with a *bap1* construct lacking the 57aa by cotransformation with ∆*VC1807*::*P_tac_-mNeonGreen,* Spec^R^ into a rugose strain lacking *rbmC* (JY071) | ZJ032 [5] |
| Δ*rbmC bap1*_Δ_*_β-prism_* | *vpvC*^W240R^*,* Δ*rbmC, bap1*_Δ_*_β-prism_,* ∆*VC1807*::*P_tac_-mNeonGreen*, Spec^R^ | Replacement of *bap1^WT^* with a *bap1* construct lacking the prism but with remaining 57aa by cotransformation with ∆*VC1807*::*P_tac_-mNeonGreen,* Spec^R^ into a rugose strain lacking *rbmC* (JY071) | ZJ087 [5] |
| Δ*rbmC bap1*_Δ_*_β-prism_*_Δ_*_57aa_* | *vpvC*^W240R^*,* Δ*rbmC, bap1*_Δ_*_β-prism_*_Δ_*_57aa_,* ∆*VC1807*::*P_tac_-mNeonGreen*, Spec^R^ | Replacement of *bap1^WT^* with a *bap1* construct lacking the β-prism domain and 57aa by cotransformation with ∆*VC1807*::*P_tac_-mNeonGreen,* Spec^R^ into a rugose strain lacking *rbmC* (JY071) | ZJ074 [5] |
| Δ*rbmC bap1*_Δ_*_57aa*(1-repeat)_* | *vpvC*^W240R^*,* Δ*rbmC, bap1*_Δ_*_57aa*(1-repeat)_,* ∆*VC1807*::*P_tac_-mNeonGreen*, Spec^R^ | Replacement of *bap1^WT^* with a *bap1* construct containing 57aa variant (1-repeat) by cotransformation with ∆*VC1807*::*P_tac_-mNeonGreen,* Spec^R^ into a rugose strain lacking *rbmC* (JY071) | XH108, This Study |
| Δ*rbmC bap1*_Δ_*_57aa*(2-repeat)_* | *vpvC*^W240R^*,* Δ*rbmC, bap1*_Δ_*_57aa*(2-repeat)_,* ∆*VC1807*::*P_tac_-mNeonGreen*, Spec^R^ | Replacement of *bap1^WT^* with a *bap1* construct containing 57aa variant (2-repeat) by cotransformation with ∆*VC1807*::*P_tac_-mNeonGreen,* Spec^R^ into a rugose strain lacking *rbmC* (JY071) | XH118, This Study |
| Δ*rbmC bap1*_Δ_*_57aa*(core motif)_* | *vpvC*^W240R^*,* Δ*rbmC, bap1*_Δ_*_57aa*(core motif)_,* ∆*VC1807*::*P_tac_-mNeonGreen*, Spec^R^ | Replacement of *bap1^WT^* with a *bap1* construct containing 57aa variant (core motif) by cotransformation with ∆*VC1807*::*P_tac_-mNeonGreen,* Spec^R^ into a rugose strain lacking *rbmC* (JY071) | XH194, This Study |
| Δ*rbmC bap1*_Δ_*_57aa*(WFFG->LGPE)_* | *vpvC*^W240R^*,* Δ*rbmC, bap1*_Δ_*_57aa*(WFFG->LGPE)_,* ∆*VC1807*::*P_tac_-mNeonGreen*, Spec^R^ | Replacement of *bap1^WT^* with a *bap1* construct containing 57aa variant (WFFG->LGPE) by cotransformation with ∆*VC1807*::*P_tac_-mNeonGreen,* Spec^R^ into a rugose strain lacking *rbmC* (JY071) | XH196, This Study |
| Δ*rbmC bap1*_Δ_*_57aa+C-terminal 57aa_* | *vpvC*^W240R^*,* Δ*rbmC, bap1*_Δ_*_57aa+C-terminal 57aa_,* ∆*VC1807*::*P_tac_-mNeonGreen*, Spec^R^ | Replacement of *bap1^WT^* with a *bap1* construct containing 57aa at C-terminus by cotransformation with ∆*VC1807*::*P_tac_-mNeonGreen,* Spec^R^ into a rugose strain lacking *rbmC* (JY071) | XH165, This Study |
| Δ*rbmC bap1*_Δ_*_β-prism+57aa*(2-repeat)_* | *vpvC*^W240R^*,* Δ*rbmC, bap1*_Δ_*_β-prism+57aa*(2-repeat)_,* ∆*VC1807*::*P_tac_-mNeonGreen*, Spec^R^ | Replacement of *bap1^WT^* with a *bap1* construct lacking the prism with remaining 57aa variant (2-repeat) by cotransformation with ∆*VC1807*::*P_tac_-mNeonGreen,* Spec^R^ into a rugose strain lacking *rbmC* (JY071) | XH122, This Study |
| Δ*rbmC bap1*_Δ_*_β-prism+57aa*(core motif)_* | *vpvC*^W240R^*,* Δ*rbmC, bap1*_Δ_*_β-prism+57aa*(core motif)_,* ∆*VC1807*::*P_tac_-mNeonGreen*, Spec^R^ | Replacement of *bap1^WT^* with a *bap1* construct lacking the prism with remaining 57aa variant (core motif) by cotransformation with ∆*VC1807*::*P_tac_-mNeonGreen,* Spec^R^ into a rugose strain lacking *rbmC* (JY071) | XH166, This Study |
| Δ*rbmC bap1*_Δ_*_β-prism+57aa*(WFFG->LGPE)_* | *vpvC*^W240R^*,* Δ*rbmC, bap1*_Δ_*_β-prism+57aa*(WFFG->LGPE)_,* ∆*VC1807*::*P_tac_-mNeonGreen*, Spec^R^ | Replacement of *bap1^WT^* with a *bap1* construct lacking the prism with remaining 57aa variant (WFFG->LGPE) by cotransformation with ∆*VC1807*::*P_tac_-mNeonGreen,* Spec^R^ into a rugose strain lacking *rbmC* (JY071) | XH146, This Study |
| Δ*rbmC bap1*_Δ_*_β-prism+C-terminal 57aa_* | *vpvC*^W240R^*,* Δ*rbmC, bap1*_Δ_*_β-prism+C-terminal 57aa_,* ∆*VC1807*::*P_tac_-mNeonGreen*, Spec^R^ | Replacement of *bap1^WT^* with a *bap1* construct lacking the prism but containing 57aa at C-terminus by cotransformation with ∆*VC1807*::*P_tac_-mNeonGreen,* Spec^R^ into a rugose strain lacking *rbmC* (JY071) | XH169, This Study |
| Δ*rbmC bap1-3XFLAG* | *vpvC*^W240R^*,* Δ*rbmC, bap1-3XFLAG,* ∆*VC1807*::*P_tac_-mNeonGreen,* Spec^R^ | 3XFLAG tagged Bap1 with *rbmC* deleted | ZJ065 [5] |
| *bap1*_Δ_*_57aa_-3XFLAG* | *vpvC*^W240R^*, bap1*_Δ_*_57aa_-3XFLAG,* ∆*VC1807*::*P_tac_-mNeonGreen,* Spec^R^ | Replacement of *bap1^WT^* with a *bap1* construct lacking the 57aa and containing a C-terminal 3XFLAG tag by cotransformation with ∆*VC1807*::*P_tac_-mNeonGreen,* Spec^R^ into a rugose strain (JY028) | ZJ036 [5] |
| Δ*rbmC bap1*_Δ_*_57aa*(1-repeat)_-3XFLAG* | *vpvC*^W240R^*, bap1*_Δ_*_57aa*(1-repeat)_-3XFLAG,* ∆*VC1807*::*P_tac_-mNeonGreen,* Spec^R^ | Replacement of *bap1^WT^* with a *bap1* construct containing 57aa variant (1-repeat) and a C-terminal 3XFLAG tag by cotransformation with ∆*VC1807*::*P_tac_-mNeonGreen,* Spec^R^ into a rugose strain (JY028) | XH106, This Study |
| Δ*rbmC bap1*_Δ_*_57aa*(2-repeat)_-3XFLAG* | *vpvC*^W240R^*, bap1*_Δ_*_57aa*(2-repeat)_-3XFLAG,* ∆*VC1807*::*P_tac_-mNeonGreen*, Spec^R^ | Replacement of *bap1^WT^* with a *bap1* construct containing 57aa variant (2-repeat) and a C-terminal 3XFLAG tag by cotransformation with ∆*VC1807*::*P_tac_-mNeonGreen,* Spec^R^ into a rugose strain (JY028) | XH198, This Study |
| Δ*rbmC bap1*_Δ_*_57aa*(core motif)_-3XFLAG* | *vpvC*^W240R^*, bap1*_Δ_*_57aa*(core motif)_-3XFLAG,* ∆*VC1807*::*P_tac_-mNeonGreen*, Spec^R^ | Replacement of *bap1^WT^* with a *bap1* construct containing 57aa variant (core motif) and a C-terminal 3XFLAG tag by cotransformation with ∆*VC1807*::*P_tac_-mNeonGreen,* Spec^R^ into a rugose strain (JY028) | XH195, This Study |
| Δ*rbmC bap1*_Δ_*_57aa*(WFFG->LGPE)_-3XFLAG* | *vpvC*^W240R^*, bap1*_Δ_*_57aa*(WFFG->LGPE)_-3XFLAG,* ∆*VC1807*::*P_tac_-mNeonGreen*, Spec^R^ | Replacement of *bap1^WT^* with a *bap1* construct containing 57aa variant (WFFG-LGPE) and a C-terminal 3XFLAG tag by cotransformation with ∆*VC1807*::*P_tac_-mNeonGreen,* Spec^R^ into a rugose strain (JY028) | XH197, This Study |
| Δ*rbmC bap1*_Δ_*_57aa+C-terminal 57aa_-3XFLAG* | *vpvC*^W240R^*, bap1*_Δ_*_57aa+C-terminal 57aa_-3XFLAG,* ∆*VC1807*::*P_tac_-mNeonGreen*, Spec^R^ | Replacement of *bap1^WT^* with a *bap1* construct containing 57aa at C-terminus and a C-terminal 3XFLAG tag by cotransformation with ∆*VC1807*::*P_tac_-mNeonGreen,* Spec^R^ into a rugose strain (JY028) | XH179, This Study |
| *bap1*_Δ_*_β-prism_-3XFLAG* | *vpvC*^W240R^*, bap1*_Δ_*_β-prism_-3XFLAG,* ∆*VC1807*::*P_tac_-mNeonGreen,* Spec^R^ | Replacement of *bap1^WT^* with a *bap1* construct lacking the prism but with remaining 57aa and containing a C-terminal 3XFLAG tag by cotransformation with ∆*VC1807*::*P_tac_-mNeonGreen,* Spec^R^ into a rugose strain (JY028) | XH024 [5] |
| Δ*rbmC bap1*_Δ_*_β-prism+57aa*(2-repeat)_-3XFLAG* | *vpvC*^W240R^*, bap1*_Δ_*_β-prism+57aa*(2-repeat)_-3XFLAG,* ∆*VC1807*::*P_tac_-mNeonGreen*, Spec^R^ | Replacement of *bap1^WT^* with a *bap1* construct lacking the prism with remaining 57aa variant (2 repeats) and containing a C-terminal 3XFLAG tag by cotransformation with ∆*VC1807*::*P_tac_-mNeonGreen,* Spec^R^ into a rugose strain (JY028) | XH123, This Study |
| Δ*rbmC bap1*_Δ_*_β-prism+57aa*(core motif)_-3XFLAG* | *vpvC*^W240R^*, bap1*_Δ_*_β-prism+57aa*(core motif)_-3XFLAG,* ∆*VC1807*::*P_tac_-mNeonGreen*, Spec^R^ | Replacement of *bap1^WT^* with a *bap1* construct lacking the prism with remaining 57aa variant (core motif) and containing a C-terminal 3XFLAG tag by cotransformation with ∆*VC1807*::*P_tac_-mNeonGreen,* Spec^R^ into a rugose strain (JY028) | XH174, This Study |
| Δ*rbmC bap1*_Δ_*_β-prism+57aa*(WFFG->LGPE)_-3XFLAG* | *vpvC*^W240R^*, bap1*_Δ_*_β-prism+57aa*(WFFG->LGPE)_-3XFLAG,* ∆*VC1807*::*P_tac_-mNeonGreen*, Spec^R^ | Replacement of *bap1^WT^* with a *bap1* construct lacking the prism with remaining 57aa variant (WFFG-LGPE) and containing a C-terminal 3XFLAG tag by cotransformation with ∆*VC1807*::*P_tac_-mNeonGreen,* Spec^R^ into a rugose strain (JY028) | XH144, This Study |
| Δ*rbmC bap1*_Δ_*_β-prism+C-terminal 57aa_-3XFLAG* | *vpvC*^W240R^*, bap1*_Δ_*_β-prism+C-terminal 57aa_-3XFLAG,* ∆*VC1807*::*P_tac_-mNeonGreen*, Spec^R^ | Replacement of *bap1^WT^* with a *bap1* construct lacking the prism but containing 57aa at C-terminus and containing a C-terminal 3XFLAG tag by cotransformation with ∆*VC1807*::*P_tac_-mNeonGreen,* Spec^R^ into a rugose strain (JY028) | XH184, This Study |
| Δ*rbmC bap1-3XFLAG* | *vpvC*^W240R^*,* Δ*rbmC, bap1-3XFLAG,* ∆*VC1807*::*P_tac_- SCFP3A,* Spec^R^ | 3XFLAG tagged Bap1 with *rbmC* deleted | XH186, This Study |
