# Supplementary material for: Conformations and sequence determinants in the lipid binding of an adhesive peptide derived from Vibrio cholerae biofilms
Source: PLoS Pathog. 2026 Feb 19;22(2):e1013990. doi: 10.1371/journal.ppat.1013990 (PMC12965690; doi:10.1371/journal.ppat.1013990)
Supplement: S2 Table — (DOCX) [file ppat.1013990.s011.docx]

| **Primer Name** | **Primer Sequence (5’ to 3’)*** | **Description** |
| --- | --- | --- |
| **Mutant constructs** | | |
| PJY140 | AATCAAACCGGGCTTTAAATTTCATCTCGAC | 3kb upstream of *bap1* |
| PJY141 | CATGATATGCAACATCTACTGAAAGAGGTGCA | 3kb downstream of *bap1* |
| PJY129 | ATATCCCGATCCAGTGCATGCAGC | 3kb upstream of *vpvc* |
| PJY130 | CCGGCTGATGCTTTGTGTCTAACGTG | 3kb downstream of *vpvc* |
| XH-U-005 | AAAACGGTTCCTTATCTAGGTGTTGAGTGGCGTACCAAAACCGTCTCTTACTCGACCACAGTACGCTATGACAT | *bap1*_Δ_*_57aa*(2-repeat)_* F |
| XH-U-006 | CCACTCAACACCTAGATAAGGAACCGTTTTAGTTTTCCACTCTAATCCTAGAGTAAACGCAGAATCTTTTGACCCC | *bap1*_Δ_*_57aa*(2-repeat)_* R |
| XH-P-041 | GGGGTCAAAAGATTCTGCGTTTACTTGGAAAACTAAAACGGTTCCTTAT | *bap1*_Δ_*_57aa*(1-repeat)_* F |
| XH-P-042 | ATGTCATAGCGTACTGTGGTCGAATAAGGAACCGTTTTAGTTTTCCA | *bap1*_Δ_*_57aa*(1-repeat)_* R |
| XH-P-045 | GTGCAACCACTGTTGATGCTCTAGGATTAGAGTGGAAAACTAAAACGGT | *bap1*_Δ_ *_β-prism+57aa*(2-repeat)_* Front F |
| XH-P-046 | ACCGTTTTAGTTTTCCACTCTAATCCTAGAGCATCAACAGTGGTTGCAC | *bap1*_Δ_ *_β-prism+57aa*(2-repeat)_* Front R |
| XH-P-047 | GGCGTACCAAAACCGTCTCTTACGTGACTGCTGACCAATCACACA | *bap1*_Δ_ *_β-prism+57aa*(2-repeat)_* Back F |
| XH-P-048 | TGTGTGATTGGTCAGCAGTCACGTAAGAGACGGTTTTGGTACGCC | *bap1*_Δ_ *_β-prism+57aa*(2-repeat)_* Back R |
| XH-P-059 | GTACCAAAACCGTCTCTTACCTAGGCCCTGAGTGGCACACTAAACAAGTGGC | *bap1*_Δ_*_57aa*(WFFG->LGPE)_* F |
| XH-P-060 | GCCACTTGTTTAGTGTGCCACTCAGGGCCTAGGTAAGAGACGGTTTTGGTAC | *bap1*_Δ_*_57aa*(WFFG->LGPE)_* R |
| XH-P-071 | CCATTCGCGTTCCGCTGAAGTATCTAGGATTAGAGTGGAAAACTAAAACGG | Amplify 57aa for C-terminus F |
| XH-P-072 | GCGGCTGGCAGAAGTATCTTTATTTCGACAGTGTCACAGGAACG | Amplify 57aa for C-terminus R |
| XH-P-073 | CGTTCCTGTGACACTGTCGAAATAAAGATACTTCTGCCAGCCGC | 57aa insertion to C-terminus F |
| XH-P-074 | CCGTTTTAGTTTTCCACTCTAATCCTAGATACTTCAGCGGAACGCGAATGG | 57aa insertion to C-terminus R |
| XH-P-079 | TCTTACTGGTTCTTTGGCTGGCACACTAAAGTGACTGCTGACCAATCACACAT | *bap1*_Δ_*_β-prism+57aa*(core motif)_* F |
| XH-P-080 | TTTAGTGTGCCAGCCAAAGAACCAGTAAGAAGCATCAACAGTGGTTGCACT | *bap1*_Δ_*_β-prism+57aa*(core motif)_* R |
| XH-P-091 | aatcaccgtcatggtctttgtagtcTTTCGACAGTGTCACAGGAACG | Amplify 57aa for C-terminus FLAG R |
| XH-P-092 | CGTTCCTGTGACACTGTCGAAAgactacaaagaccatgacggtgatt | 57aa insertion to C-terminus FLAG F |
| XH-P-115 | TCTTACTGGTTCTTTGGCTGGCACACTAAATCGACCACAGTACGCTATGACAT | *bap1*_Δ_*_57aa*(core motif)_* F |
| XH-P-116 | TTTAGTGTGCCAGCCAAAGAACCAGTAAGAAGTAAACGCAGAATCTTTTGACCCC | *bap1*_Δ_*_57aa*(core motif)_* R |
| ZJ-P-019 | CTTGTCATCGTCATCCTTGTAATCGATA | Universal sequencer for 3xFLAG |
| ZJ-P-026 | AGCAGCATTTTGAAAACTTCCGC | 2.7kb upstream of *bap1* |
| ZJ-P-027 | ATGAAATTCACGATAACCAGAAAACCG | 2.7kb downstream of *bap1* |

**References for Supplementary Files**

1. Yan J, Moreau A, Khodaparast S, Perazzo A, Feng J, Fei C, et al. Bacterial biofilm material properties enable removal and transfer by capillary peeling. Adv Mater. 2018 Nov 1;30(46):1804153.

2. Hollenbeck EC, Fong JCN, Lim JY, Yildiz FH, Fuller GG, Cegelski L. Molecular determinants of mechanical properties of *V. cholerae* biofilms at the air-liquid interface. Biophys J. 2014;107(10):2245–52.

3. Kaus K, Biester A, Chupp E, Lu J, Visudharomn C, Olson R. The 1.9 Å crystal structure of the extracellular matrix protein Bap1 from *Vibrio cholerae* provides insights into bacterial biofilm adhesion. J Biol Chem. 2019;294(40):14499–511.

4. Yan J, Sharo AG, Stone HA, Wingreen NS, Bassler BL. *Vibrio cholerae* biofilm growth program and architecture revealed by single-cell live imaging. Proc Natl Acad Sci USA. 2016 Sep 6;113(36):e5337-5343.

5. Huang X, Nero T, Weerasekera R, Matej KH, Hinbest A, Jiang Z, et al. *Vibrio cholerae* biofilms use modular adhesins with glycan-targeting and nonspecific surface binding domains for colonization. Nat Commun. 2023 Apr 13;14(1):2104.

6. Zhang Q, Li J, Nijjer J, Lu H, Kothari M, Alert R, et al. Morphogenesis and cell ordering in confined bacterial biofilms. Proc Natl Acad Sci USA. 2021 Aug 3;118(31):e2107107118.
