# Supplementary material for: Conformations and sequence determinants in the lipid binding of an adhesive peptide derived from Vibrio cholerae biofilms
Source: PLoS Pathog. 2026 Feb 19;22(2):e1013990. doi: 10.1371/journal.ppat.1013990 (PMC12965690; doi:10.1371/journal.ppat.1013990)
Supplement: Attachment — Submitted filename: Response to Reviewers.pdf [file ppat.1013990.s014.pdf]

Dear Editors of *PLOS Pathogens*,

Thank you very much for handling our manuscript. We appreciate the comments and constructive suggestions of all three reviewers.

Following the reviewers' comments, we have made significant revisions to the manuscript, including additional experimental and bioinformatic results. We have also added 2 new panel to the main figures and 3 new SI figures in response to the reviewers' requests. Point-by-point responses to the reviewers' comments are listed below, where the reviewers' comments are in black, and our responses are in blue.

We hope that you now find the manuscript acceptable for publication in *PLOS Pathogens*. In addition to this response letter, please find attached the revised main text and revised supplementary information. We have highlighted all major changes in the text in red.

Sincerely,

on behalf of all the authors,

Jing Yan

Reviewer #1: In this manuscript by Huang et al, the authors investigate the role of a peptide domain of Bap1 in mediating lipid binding. Using a combination of in vitro, in vivo, and in silico approaches, the authors demonstrate that a central aromatic-rich motif is sufficient for membrane binding, by forming a beta-hairpin that inserts into the membrane bilayer. Adjacent regions do not insert into the membrane, but still contribute to lipid binding via enhanced avidity.

Overall, I found this study to be very well executed and the conclusions are well supported. I do not have any major concerns, but offer a few suggestions as described below.

We thank the reviewer for the positive comments and for appreciating our work.

Reviewer #1: 1) The authors made good use of various mutations in the peptide loop motif to determine the number of repeats required for efficient binding. Given that the four pseudo repeats are not identical, it might be worthwhile to see if they have different binding properties.

We agree with the reviewers that delving into the difference between the four pseudo repeats will be an interesting subject. Since they are likely all close to the negative control in our adhesion assay if we study them as four independent short peptides, we will have to generate four peptides that essentially have four identical repeats. This would require significant efforts, and we will defer the experiment to a future study.

2) The authors point out that this peptide sequence is conserved amongst other Vibrio species. Is it found outside of Vibrio? If so, could you please describe this conservation in the text?

Our searches indicate that this peptide is restricted to the Vibrio genus. We queried the NCBI non-redundant (nr) protein database using BLASTP and required 100% identity and 100% coverage. All returned hits were from Vibrio species. We have clarified this point in the manuscript on lines 360-362: “A BLASTP search of the sequence against the NCBI non-redundant (nr) protein database recovered only Vibrio sequences with 100% identity and 100% coverage, indicating that this peptide is Vibrio-restricted. This sequence was also found exclusively in the Bap1 proteins, highlighting its distinctive role”.

3) Given that this paper is quite multidisciplinary, some readers may have difficulty understanding all of the approaches. The authors could add additional details/explanations to make it more accessible. For example, in Figure 4D, the y-axis is labeled "contact frequency" but this is not explained. I assume this is contact frequency between an amino acid and the membrane surface, but it's not clear. Similarly, Figures 4B-C show secondary structure frequency. The idea is that beta-turns are stabilized in the lipid bilayer. I see that interpretation based on the orange bars, but the legend says that these are anti-parallel beta-sheets. Indeed, it doesn't seem that any of the colors refer to beta-turns. This needs to be clarified.

We thank the reviewer for providing this perspective. Indeed, in Figure 4D, the contact frequency refers to the contact frequency of an amino acid with the membrane surface. In Figure 4B, the two anti-parallel β-sheets adjacent to each other signify a tight β-turn structure.

We have updated the explanation in the description of the simulation results. We have also tried to provide additional explanations throughout the text whenever possible.

4) Figure S6 is a very interesting result. I'm not sure why that is in the Discussion. It seems that this should be moved up to the results section.

We thank the reviewer for the suggestion. We have moved the figure to the main text. We have also repeated the experiment and improved the results. See the updated main Fig. 6.

Minor typos:

1) Line 35: capable of forming "biofilms". Fixed.
2) Line 121: "ratios)", modeling the plasma membrane. Fixed.

Reviewer #2: The manuscript by Huang, Prasad, et al. is focused on addressing the mechanism by which a previously identified biofilm protein binds to lipid membranes. Specifically, prior work identified a 57 amino acid loop within the Bap1 protein that is necessary for lipid binding. And the aim of this study was to systemically characterize the features of this 57 amino acid loop that are critical for lipid binding. This is a timely and important topic that is broadly relevant for the fields of bacterial pathogenesis and biofilm formation. The manuscript is also well-written and clearly presented. One of the central conclusions from the manuscript is that there is a core motif within the 57 amino acid Bap1 loop - SYWFFGWHTK - that is critical for lipid binding. In particular, they hypothesize that the WFFG residues at the center of this motif is critical for inserting into membranes. While some of the data presented support this conclusion, important controls and critical experimental variants to rigorously test this claim, are missing. Thus, the conclusions need to be better qualified and softened, or additional supporting evidence should be provided to support the model presented.

We thank the reviewer for recognizing the importance of our work, and the overall positive assessment of our work. We have provided additional results to support our claims, and also softened some of the conclusions/claims according to the reviewer's suggestion.

Major comments:

The results suggest that multiple regions of Bap1 facilitate lipid binding – including the pseudo repeats, the WFFG beta-hairpin, and possibly the beta-prism domain. The experiments presented, however, do not rigorously dissect this redundancy to support the model presented. In Fig. 2C, it is shown that the core motif is sufficient to bind lipids. And a major conclusion of the manuscript is that the central WFFG residues within the core motif are critical for inserting into lipid membranes. But a variant of the core motif where the WFFG residues are mutated (WFFG -> LGPE) is not tested to demonstrate that these central residues are critical. Instead, these residues are mutated within the longer 57 amino acid loop. But this only shows a very

modest ~5-fold reduction in lipid binding. This observation is attributed to the ability of the pseudo repeats to bind lipids in the absence of the central WFFG residues. Thus, the importance of the WFFG residues within the core motif is only indirectly supported by the evidence provided. To demonstrate the critical importance of these residues for lipid binding by the core motif, the authors should test a SYLGPEWHTK peptide for lipid binding. If their model is correct, this peptide should resemble the FITC-dextran control.

On a related note, Fig. 2g shows that the core motif is sufficient to bind lipids in their biofilm-based assay. Testing the variant where the central WFFG residues are mutated in the "core motif" construct would strongly support their claim. Especially in this more physiological relevant assay.

We thank the reviewer for pointing out this important control. We have chemically synthesized the control peptide with the suggested sequence (SYLGPEWHTK) labeled with FITC and repeated the adhesion assay. Indeed, we found that it adheres *minimally* to lipid-coated beads (Fig. S4). We have measured the CD spectrum of the control peptide missing the WFFG (SYLGPEWHTK); the 205 nm peak is absent, indicating the disruption of the β-turn structure. The corresponding results are now included in Figure 3c and Figure S4.

Considering that the *in vitro* and biofilm assays generate matching results in general, we expect that the SYLGPEWHTK mutant will also be defective in the SUV-capturing assay with biofilms. The other proposed experiment on testing the mutant sequence in the biofilm assay is thus largely confirmatory, yet it requires substantial time and resources to make and validate the mutant and perform the assay, all of which are not trivial. Unfortunately, given the logistical constraints we are facing now, we are unable to test this sequence variant in the biofilm assay.

Line 220-223: It is not clear where the concern about the beta-prism domain came from. Fig. 2g shows that the Bap1 variant lacking the 57 amino acid loop lacks the ability to recruit lipids. So, it does not appear to be sufficient to bind lipids. Regardless, this background is used to test the effect of the central WFFG residues on lipid binding, however, the potential redundancy with the pseudo-repeats results in only a very modest <2-fold decrease in lipid binding. Again, this only provides indirect support for the critical role that these residues play in lipid binding within the core motif.

In our previous publication (*J. Biol. Chem.*, 294, 14499), we showed that the region of the β-prism domain next to the 57 amino acid loop is positively charged and has some aromatic residues (see Figure R1 below) – a similar feature to the 57aa peptide. This led us to make the earlier suggestion "…the β-prism's cap together with the 57aa loop may form a continuous, positively-charged and aromatic surface enabling Bap1 to adhere…" (*Nat. Commun.*, 14, 2104). Results in Fig. 2g are from the genetic background with the β-prism present and results from Figure S6d are from the genetic background with the β-prism deleted. Despite the concern, the mutant analysis gives largely consistent results in both backgrounds, except for one mutant; see legend in Fig. 6S for more detailed discussions.

We agree that the difference we see is small with the pseudo-repeats (WFFG->LGPE), which confirms that the pseudo-repeats are also capable of binding to SUVs in the biofilm context through the avidity effect detailed in the manuscript. Note that in the biofilm context, the biofilm surfaces are covered with Bap1 and thus the 57aa loop; therefore, there could potentially be many 57aa copies at the SUV-biofilm interface. We have updated the text accordingly.

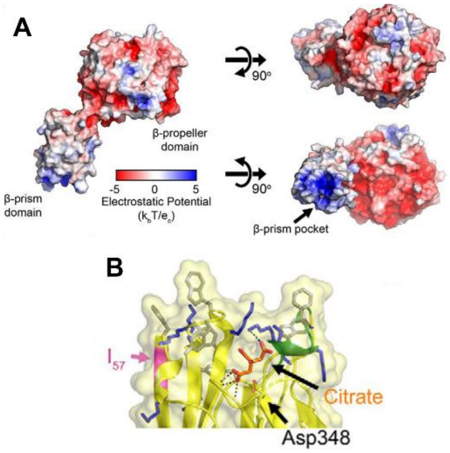

Fig. R1: Surface characteristics of the β-prism domain. (**A**) Electrostatic surface potential of Bap1 calculated from the crystal structure. Note that the 57aa loop had to be removed for crystallization due to its stickiness and disorder. (**B**). A closer look at the cap of the β-prism domain with aromatic and lysine residues highlighted in *gray* and *blue stick* representation, respectively. The location of the 57aa insertion is shown in magenta.

In Fig. 3c-d, the CD spectral analysis suggests that the core motif forms a beta-hairpin. But no meaningful controls are presented. A mutant of the core motif or 57 amino acid loop where the central WFFG residues are mutated should be tested. According to the model presented, these mutations should prevent formation of the beta-hairpin.

We thank the reviewer for the excellent suggestion. We have measured the CD spectrum of the control peptide SYLGPEWHTK where the central WFFG of the core motif is replaced with LGPE; the 205 nm peak is absent, indicating the disruption of the β-turn structure. The corresponding data is added to Fig. 3c.

In Fig. 5, it does not look like the core motif is binding to the surface of cells as indicated in the text. The signal appears to be within intracellular vesicles. This looks more like the Caco-2 cells are simply ingesting the peptide. Can the authors rule out this possibility?

Thanks for noticing this apparent localization difference. To show better surface association, we used FM 4-64 as the membrane stain instead of actin stain, and we updated Figure 5 accordingly. The original figure is now included as Fig. S8. We do see clear co-localization of FM 4-64 signal and the core-motif peptide, suggesting membrane association. We cannot rule out the possibility of the peptides being ingested, but we do not see any such cell-associated

signals in the negative controls or in the 1- or 2-repeat, which is the main point of the figure. We have modified the text accordingly.

Minor comments:

Line 160-162: It should be specifically stated that it is the “FITC fluorescence” signal that should be proportional to the number of peptide molecules bound. Related to this, it is unclear whether all of the tested lipids are coating the silica beads equally. Because Rhodamine labeled lipids are used, a plot of the rhodamine fluorescence in each sample would be a valuable control to include. It is also not clear what is meant by “excess fluorescence”. Is this just the fluorescence above background?

We have now stated that FITC fluorescence signal should be proportional to the number of peptide molecules bound. We have shown in the new Figure S3 that lipid-coating is homogenous and similar for different lipid compositions. We also clarified that excess fluorescence means the surface fluorescence minus the background signal in the solution. Note that the solution signal is different for each peptide concentration. We use this quantification following the thermodynamic definition of adsorption (Israelachvili, Intermolecular and Surface Forces, 3rd Ed.).

Line 334-336: It is not clear whether this conservation is reflective of importance of these residues or just a limited amount of sequence divergence among homologs. What is the conservation of residues within the 57 amino acid loop compared to the remainder of the Bap1 protein? If residues within the loop are more conserved than the rest of the protein, this would help support the stated conclusions.

Thank you so much for the suggestion. We performed the analysis, and we found there is no significant difference in the conservation of residues in the 57aa loop compared to the residues in other parts of the Bap1 protein (see Figure R2 below), likely due to the high conservation observed in some of the other parts of Bap1 due to their key functions. See more discussions below.

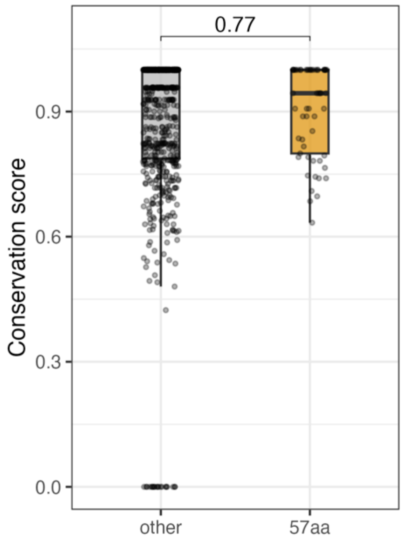

Fig. R2: Conservation score of the 57aa loop compared to the rest of Bap1.

Fig. 6c – it is unclear how evolutionary coupling analysis could be informative in this case because the authors are showing complete conservation of the residues in question in Fig. 6b. Admittedly, this is not my area of expertise, but I would appreciate some clarification from the authors on this point.

The EVcouplings analysis addresses a different question from residue conservation. While conservation measures how well individual positions are preserved across homologs, EVcouplings analysis detects pairs of positions that co-vary during evolution, meaning that a substitution at one site is often accompanied by a compensatory change at another. Such co-variation suggests that the residues are functionally or structurally linked. In our case, although the 57aa loop is generally conserved, substitutions still occur, and EVcouplings analysis identifies coupled residue pairs within this region. We revised the text on lines 377-378 to clarify this: “We also conducted EVcouplings analysis to detect pairs of positions that co-vary during evolution”.

Fig. S6 and S7 – it is fairly unconventional for data to be presented in the Discussion section. The explanation of these data should be moved to the Results section.

We have now presented the original Figure S6 as Main Figure 6, following the reviewer’s suggestion, and added a new section accordingly. We have decided to leave the original Figure S7 in the SI, since it is a tangential point. We found it hard to put this piece of data in the main text without disrupting the flow of the paper.

Reviewer #3: This study by Huang et al. investigates how a 57–amino acid loop in the Vibrio cholerae biofilm-associated protein Bap1 enables binding to lipids and abiotic surfaces. The

authors apply a well-designed orthogonal toolkit to address this question. Overall, the study is very nicely executed and clearly written. I do, however, have a few suggestions that could help strengthen the model and provide further clarity in the text.

We thank the reviewer for the encouragement and the positive review.

Major Comments:
Figure S3A – The bap mutant shows a phenotype distinct from the 57aa loop mutant, but it was not immediately clear why these mutants did not phenocopy each other. Similarly, the rationale for performing experiments in the rbmC background was not clearly explained. Please clarify these points in the text to guide interpretation.

In our previous publication (*J. Biol. Chem.*, 294, 14499), we showed that the region of the β-prism domain next to the 57aa loop is positively charged and has some aromatic residues – the similar features that the 57aa also has. See Figure R1 above in response to reviewer 2. These exposed hydrophobic residues also contribute to the hydrophobicity of the biofilm, and that is why the Δ*bap1* and $bap1_{\Delta 57aa}$ mutants do not phenocopy each other. In fact, this is consistent with the observation that the $bap1_{\Delta\beta\text{-}prism}$ mutant also has a non-zero contact angle. When both are deleted (i.e. the $bap1_{\Delta\beta\text{-}prism\ \Delta 57aa}$ mutant in the 4th column of Fig. S5b), the contact angle goes to ~ 0, similar to the Δ*bap1* negative control. We have added this discussion to the caption of Figure S5. Note that we have updated the nomenclature of the mutants to make them easier to understand.

We use the Δ*rbmC* background because RbmC, another partially redundant biofilm adhesin in *Vibrio cholerae*, has two β-prisms (but no 57aa) that can potentially confound the experiments, for the same reason. The confounding effect is well documented in our earlier work (Huang *et al.*, *Nat. Commun.* 14, 2104). For consistency and simplicity, we decided to use the Δ*rbmC* strain background. We have updated the caption of Figure 2g to clarify these points.

Lines 164-167 – This section is written too concisely. Although several lipid compositions are tested, it would help the reader if the rationale for their were explained more explicitly. It would also be useful to specify whether the tested lipids are typically found in the inner or outer leaflet.
Figure 2B – Please double-check the labeling. The legend specifies DOPS and DOPC, but these are not visible in the figure itself.

We thank the reviewer for the great suggestion. We have now included the reasoning for each lipid included in the study (see Line 166-172). We have also updated the figure label to DOPC/DOPS to be more precise.

It would also be helpful to include a representative image of the adsorption assay. In addition, in the legend or main text, please clarify that non-adhered material was washed away, as this was only evident from reading the methods section.

We have included representative images of the adsorption assay in Fig. S3. We have also updated the schematic figure in Fig. 2e to reflect the fact that the non-adhered SUVs were washed away. We have updated the main text and legends accordingly.

244-246- Have the authors considered whether modifying the hydrophobicity of the reaction buffer might influence binding profiles? Even if not tested directly, a short comment on this possibility would help contextualize the model.

We thank the reviewer for the suggestion; we added a sentence in the discussion (Line 445-447) to help contextualize the model.

Figure 6A – Please provide some numbers be provided for how many species did or did not contain the bap loop?

Among the 210 Vibrio species we analyzed, only 7 species (3.3%) contain the Bap1 loop. We have modified the text on lines 365-367 to: "The 57aa loop is present among Bap1 proteins from seven *Vibrio* species out of the 210 species we investigated, including *V. anguillarum*, *V. ordalii*, *V. metoecus*, and *V. cholerae*, with the majority (63%) of the sequences originating from *V. cholerae*."

The two distinct lineages identified are fascinating; have the authors examined whether one lineage is enriched in human or pandemic V. cholerae strains?

In our previous study (*mSystems*, 10:e00060-25), we addressed this question and found that *Vibrio cholerae* species can be further divided into seven distinct subgroups or clades. Notably, strains possessing loop-less Bap1 proteins are predominantly enriched in two of these seven clades, none of which include pandemic *V. cholerae* strains. In contrast, all pandemic and pandemic-like strains are concentrated in clades lacking loop-less Bap1 proteins, particularly clade 5.

A caveat to note is that ~80% of the genomes analyzed are from V. cholerae, which may skew the results. This limitation should be acknowledged in the text.

Thanks for raising this concern. We have tried to minimize the oversampling effect from *V. cholerae* by using non-redundant sequences to build the gene tree and corresponding text has been modified in the Methods: "Finally, a codon-based phylogenetic Bap1 encoded gene tree containing 392 non-redundant sequences was built …" Furthermore, we acknowledged the limitations in the Discussions (lines 461-466): "In addition, because the taxonomic distribution of Bap1 is intrinsically narrow, and despite our inclusion of all GTDB genomes relevant to this gene family, current genomic datasets are inevitably skewed toward *V. cholerae* due to existing research and sequencing biases. This limitation is largely inherent to the available data. As additional genomes from underrepresented taxa become available, a clearer and more comprehensive view of the evolutionary patterns of Bap1 will likely emerge."

In cases where a bacterium carries two copies of Bap (one with the 57aa loop and one without), is there any expected functional consequence for biofilm formation? If this is not yet known, a brief comment on how such cases might be interpreted would be valuable. Depending on feasibility, it may also be an interesting experiment to consider, though I will add that it is not strictly necessary to do.

We understand the reviewer's concern and appreciate the comment. We agree that experimental validation would provide a clearer understanding of the consequence for biofilm formation of *V. cholerae* strains carrying two copies of Bap1 genes. We have obtained this strain and made an attempt. However, due to challenges with the strain's growth and biofilm formation tests in the lab, obtaining such data requires additional trials and a significant time investment. As an alternative approach, we have analyzed the genes exclusively present or absent in Vibrio strains with loop-less Bap1 to briefly explore their potential roles in metabolic pathways in our previous study (*mSystems*, 10:e00060-25).

Minor Comment:
195-197 – There is inconsistency in fluorescent protein nomenclature. Some places mention mNeonGreen, while the legend in panel f lists Cfp. Please clarify.

The labeling is correct. In most of our experiments, we use mNeonGreen, which is the brightest fluorescent protein in *V. cholerae* we have in hand. For the particular experiment in Fig. 2f, we had to use the Anti-FLAG antibody conjugated to FITC (green) to label the Bap1 matrix, so we were forced to use a cyan fluorescent protein SCFP3A for this particular experiment. We have updated the caption to clarify.
