# Supplementary material for: Conformations and sequence determinants in the lipid binding of an adhesive peptide derived from Vibrio cholerae biofilms
Source: PLoS Pathog. 2026 Feb 19;22(2):e1013990. doi: 10.1371/journal.ppat.1013990 (PMC12965690; doi:10.1371/journal.ppat.1013990)
Supplement: Attachment — Submitted filename: Response to Reviewers.docx [file ppat.1013990.s015.docx]

Dear Editors of *PLOS Pathogens*,

Thank you very much for handling our manuscript. We have integrated all changes suggested by the reviewers. We hope that you now find the manuscript acceptable for publication in *PLOS Pathogens*. In addition to this response letter, please find attached the revised main text with tracked changes.

Sincerely,

on behalf of all the authors,

Jing Yan

**Reviewer #1:** I have one minor suggestion regarding data presentation. One of the reviewers pointed out that the assays showing that the peptide binds to Caco2 cells are unclear, regarding whether the peptide is on the cell surface or in internalized vesicles. Since these images were acquired by confocal microscopy, could you add a cross-section of the z-stack to Figure S8 to clearly show the peptide on the surface?

We thank the reviewer for the suggestion. In the updated Figure S8 (panel b and c), we have included the cross-sectional view of various peptides binding to Caco2 cells. As the data show, for WT and WFFG->LGPE peptides, the signals are clearly associated with the cell membranes. For the core motif, the localization is less obvious but the FITC signals are still cell associated. To be consistent, in line 330 in the main text, we changed “Figure 5 and Figure S8 show that WT, WFFG→LGPE, and core motif peptides show strong cell-membrane-associated signals” to “… cell-associated signals”.

Line 167: I think this should be "phosphatidylcholine". Fixed.
Line 169: "phosphatidylserine". Fixed.
Line 183: I would change the text to "mutating these residues". As I understood the text, the central residues were changed, not removed. Fixed.
